# Characterization of a Chimeric Resilin-Elastin Structural Protein Dedicated to 3D Bioprinting as a Bioink Component

**DOI:** 10.3390/nano14090749

**Published:** 2024-04-25

**Authors:** Violetta Cecuda-Adamczewska, Agnieszka Romanik-Chruścielewska, Katarzyna Kosowska, Natalia Łukasiewicz, Iwona Sokołowska, Paulina Korycka, Katarzyna Florys-Jankowska, Agnieszka Zakrzewska, Michał Wszoła, Marta Klak

**Affiliations:** 1 Foundation of Research and Science Development, 01-424 Warsaw, Poland; aga.romanik@wp.pl (A.R.-C.); katarzyna.kosowska@fundacjabirn.pl (K.K.); natalia.lukasiewicz@fundacjabirn.pl (N.Ł.); iwona.sokolowska@fundacjabirn.pl (I.S.); korycka.paulina93@gmail.com (P.K.); katarzyna.florys.jankowska@fundacjabirn.pl (K.F.-J.); 2 Polbionica Ltd., 01-424 Warsaw, Poland; agnieszka.zakrzewska@polbionica.com (A.Z.); michal.wszola@polbionica.com (M.W.)

**Keywords:** recombinant proteins, resilin- and elastin-like engineered polypeptide, cytotoxicity, 3D bioprinting

## Abstract

In this study we propose to use for bioprinting a bioink enriched with a recombinant RE15mR protein with a molecular weight of 26 kDa, containing functional sequences derived from resilin and elastin. The resulting protein also contains RGD sequences in its structure, as well as a metalloproteinase cleavage site, allowing positive interaction with the cells seeded on the construct and remodeling the structure of this protein in situ. The described protein is produced in a prokaryotic expression system using an *E. coli* bacterial strain and purified by a process using a unique combination of known methods not previously used for recombinant elastin-like proteins. The positive effect of RE15mR on the mechanical, physico-chemical, and biological properties of the print is shown in the attached results. The addition of RE15mR to the bioink resulted in improved mechanical and physicochemical properties and promoted the habitation of the prints by cells of the L-929 line.

## 1. Introduction

One of the fundamental problems with 3D printing for biomedical purposes is the definition of the bioink components and the matching of their physicochemical, biochemical, or biological properties to the expectations of the resulting construct [1,2,3]. A major goal in bioprinted models for tissue engineering is to mimic the properties of native tissues and organs as closely as possible [4,5,6]. For this purpose, in tandem with proteins derived from tissue decellularization (dECM), other substances such as collagen, gelatin hyaluronic acid, alginate, cellulose, or chitosan [7] are supplemented to the hydrogels tested in 3D bioprinting. Each can slightly affect the mechanical and biochemical properties of both solutions and printed structures. To modify their properties, the components of the bioink are enriched chemically, e.g., in a process to allow cross-linking of the material under UV light. Bioinks can also be modified by the addition of other bioactive components (fibronectin, laminin, glycosaminoglycans, and growth factors) [7] to improve the interaction of cells with the inhabited biomaterial. As a result, an appropriately selected supplement can alter the cells’ microenvironment, creating favorable conditions for adhesion, proliferation colonization, and in the end, the cell expansion.

In many cases, good printability does not go hand in hand with adequate features of the material, or conflicts with a spatial structure that is conducive to being inhabited by living cells [8]. ECM derived from organ or tissue decellularization is an expensive material and its composition varies from donor to donor and is therefore difficult to standardize, making the use of natural sources of ink components such as dECM (extracellular matrix) problematic. Detergents may be used in the tissue decellularization process [9], and effective rinsing of the resulting dECM is also critical, as detergent residues may adversely affect bioink properties [10,11]. In particular, the presence of detergents [12,13] may not only impact on cell colonization and growth in the print [14], but even be cytotoxic to surrounding tissue [15,16]. Defining reproducible printing parameters with dECM containing bioinks is therefore difficult. In this context, the effort to obtain reproducible and well-described bioink components is not only important for implementation but also for cognitive reasons. The creation of recombinant peptides and proteins modeled on naturally occurring structures is one of the many proposed solutions. Examples of such model proteins are collagen, elastin, silk, or resilin, which are well known for their structural functions and their mechanical properties [17,18]. Derivative sequences or entire domains from these proteins are used to design innovative recombinant proteins called hybrid or chimeric proteins, resembling a monster [18,19] known from Greek mythology. Depending on the fragments used and their detailed composition, the designed proteins are expected to have high strength (collagen or silk domains) and/or elastic properties (elastin, resilin, spider silk).

Due to their unique mechanical and biological properties, the elastomeric proteins, elastin and resilin, have been used extensively in the development of biomaterials for medical applications [20,21] for more than the last twenty years. Elastin is known as one of the major components of the extracellular matrix. However, elastin biological function is not limited to this task; it also plays a role in modulating cell behavior and promoting tissue repair [22]. Resilin, first discovered in locust wing-hinges [23] and found in many insects and other arthropods, is a disordered natural extracellular protein. The specific structure allows both elastin and resilin to undergo large deformations without breaking and to return to their original shape when the source of stress is removed [24]. Nevertheless, depending on the proportion of domains from these two proteins, the properties of the final hybrid protein are difficult to predict. For example, depending on the length of the resilin and elastin inserts and thus on the ability to self-assemble spherical or cylindrical micelles, the resilin-elastin copolypeptides obtained by the groups of Chilkoti et al. [25] and Bracalello et al. [18] showed different upper and lower critical solution temperatures (UCST and LCST, respectively).

The recombinant hybrid protein described in this paper was obtained by genetic engineering. It was produced in a prokaryotic *Escherichia coli* expression system. The protein produced from the bacterial culture is composed of the structural protein domains of resilin and elastin. A putative elastic repeat motif from the resilin amino-terminal domain of Drosophila melanogaster described by the sequence GGRPSDSYGAPGGGN [26] was used. Elastin structure of mammalian elastin consists among others of pentapeptide repeats VPGXG, where X, referred to as the guest residue, is any natural amino acid other than L-proline [27]. Elastin-like domains are usually represented by the sequences VPGXG (where X = V, I, A) in elastin-like polymers (ELPs), and the VPGIG and VPGAG variants have been chosen and designed for this research protein construct. The recombinant hybrid protein that is the subject of this work is further enriched in functional domains. First, the RGD motif of the fibronectin cell adhesion sequence AVTGRGDSPASS [28] and the extracellular matrix metalloproteinase (MMP-2) recognition motif GPQGIWGQ [29,30,31] were used. Enrichment of recombinant hybrid protein sequences with fibronectin-derived integrin-binding RGD domains promotes adhesion of many types of endothelial cells, smooth muscle cells, and fibroblasts [32,33,34,35], so that recombinant protein-enriched biomaterials promote growth of cells in physical contact. Metalloproteinase-sensitive MMP sequences derived from the human alpha(I) collagen chain have been attached to recombinant structural proteins to promote proteolytic degradation associated with the ability of proliferating cells to rearrange the extracellular matrix. In addition, the engineered molecule contains a lysine-rich K+ cross-linking domain to enable chemical modification of recombinant structural proteins (GGKGGKGGKGG). The presence of the K+ motif is intended to functionalize the peptides with selected chemical groups to facilitate controlled cross-linking of recombinant hybrid protein-enriched biomaterials, desirable for bioprinting. Using the described domain combinations, the developed protein can be used in the remodeling of polymeric biomaterials by cells in the colonization, migration, proliferation, and growth of new tissues. The resulting recombinant hybrid proteins can modulate cell growth by tuning RGD density and introducing MMP-sensitive domains into the hydrogels to simulate the extracellular matrix (ECM).

There are a number of bioprinting techniques, such as material extrusion, direct energy deposition, powder bed fusion, sheet lamination, material spraying, binder spraying, and VAT photopolymerization [36]. Each of the above-mentioned techniques has a number of advantages and limitations mainly related to the characteristics of the materials used. In their research paper [37], the authors characterized the extrusion printing technique using commercially available, commonly used bioink materials. It was shown that the materials used for printing biotechnology models should exhibit, in addition to appropriate mechanical parameters, biocompatibility with cellular material and good printability. A reproducible two-step procedure for evaluating printability was proposed, guaranteeing the screening of materials capable of forming stable fibers and 3D structures. A mathematical model was proposed that predicts the perimeter of printer parameters using materials with certain rheological properties. The article demonstrates the necessity of high flexibility of the material used for extrusion printing. Among the various bioprinting techniques, the droplet jet (DOD) approach to bioprinting facilitates non-contact deposition of droplets of materials and cells in small volumes to achieve optimal cell–matrix and cell–cell interactions. The study showed that the higher viscoelasticity of bioink helps stabilize droplet fibers. A significant increase in the viscosity of the bioink allows droplets to be deposited on the wetted surface of the substrate in the absence of splashing and has significantly improved the accuracy of droplet deposition. Understanding the impact of bioink properties (e.g., viscoelasticity and bio-liquid viscosity) on printing efficiency and cell proliferation is important for the formulation of new bioink [38]. Vat-polymerization bioprinting is based on light curing and enables computer-aided modeling of 3D cell-filled structures in a spot, layer-by-layer or volumetric manner, using vats filled with photoactive bioresin(s). A good example is a research work [39] presenting a methodology for 3D printing based on vat polymerization from the perspective of hardware, software, and bioresin selection.

**Figure 1:**
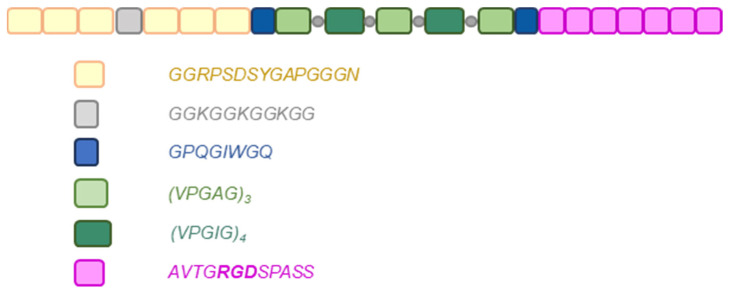
The structure of the RE15mR hybrid protein (294 aa). Consecutive blocks indicate the resilin (yellow), cross-linking (grey), cuts for metalloproteinase (blue), elastin (green), and the sequence containing the fibronectin RGD motif (pink) domains. Grey circles indicate the insertion sites of additional lysine residues beneficial for cross-linking process.

To sum up, the recombinant RE15mR hybrid protein described in this paper is sequentially composed of three resilin domains, one K+ domain, three resilin domains, one MMP domain, three elastin domains, four elastin domains, three elastin domains, four elastin domains, three elastin domains (elastin sequences were separated by lysine residues), one MMP domain, and seven RGD domains. Figure 1 shows a schematic structure of the recombinant RE15mR protein. The amino acid sequence of RE15mR contains 13% proline and 34% glycine residues. The complete amino acid sequence of the RE15mR protein is available in the Supplementary Materials. The molecular mass of the resulting full-length protein is approximately 26 kDa. By introducing sequences with a cleavage site for the metalloproteinase, it is possible to modify the size of the protein molecules in the printed model. The RGD sequences are intended to provide a friendly environment for cell colonization and growth. The synthetic recombinant protein RE15mR can be successfully used in many applications, including as an addition to biomaterials used in 3D bioprinting technology, thus improving their functional properties, such as viscosity, printability, or strength, as well as biocompatibility and biodegradability.

## 2. Materials and Methods

### 2.1. Design DNA Construct

In the experiments carried out, a pET11a plasmid vector containing a T7 phage-derived promoter, into which the sequences encoding the recombinant hybrid protein RE15mR (gene synthesis service, GeneScript Biotech, Rijswijk, The Netherlands) were cloned, was designed as a gene carrier. The nucleotide sequence of the recombinant protein gene was optimized for expression in *E. coli* cells. An expression vector was designed to express hybrid protein efficiently and stably in BLR(DE3) cells. A map of the expression vector encoding the recombinant RE15mR hybrid protein is provided in the Supplementary Materials.

### 2.2. Polypeptide Expression

Competent E. coli cells of strain BLR(DE3) (Novagen, Merck, Darmstadt, Germany) were transformed with plasmid RE15mR-pET11a and grown in flasks in 500 mL volume of LB medium supplemented with antibiotic (ampicillin 50 mg/L) at 30 °C on a microbial shaker at 150 rpm until an optical density of OD_600_ 0.7–0.9 was reached. Expression of the recombinant protein was induced by the addition of isopropyl-β-D-1-thiogalactopyranoside (IPTG) at a concentration of 0.2 mM. The culture was continued for 6 h at 37 °C with shaking at 200 rpm until the optical density of the culture reached approximately OD_600_ 3.

### 2.3. Protein Purification

After completion of the culture, the bacterial biomass was recovered from the culture medium by centrifugation. The bacterial cells obtained were resuspended in a lysis buffer consisting of 0.5 M NaCl, 50 mM Tris-HCl pH 7.5, 10 mM EDTA pH 8.0, and 5 mM β-mercaptoethanol with protease inhibitors (0.35 mg/mL lysozyme, 0.7 mM PMSF, and 10 mM benzamidine). Suspension was sonicated on ice at 33% amplitude for 10 × 10 s (SONOPULS sonicator, sonotrode KE76, Bandelin, Berlin, Germany) to disintegrate cell structures and release intracellular proteins. To precipitate the remaining bacterial DNA in solution, 0.4% (*w*/*v*) polyethyleneimine (PEI, Sigma-Aldrich, St. Louis, MO, USA) was added to the suspension. Centrifugation was used to separate the insoluble fractions of proteins, non-integrated bacterial cells and precipitated DNA from the supernatant containing the RE15mR protein. The recombinant protein supernatant was then incubated at 90 °C for 20 min and the denatured proteins centrifuged. A protease inhibitor mixture (cOmplete, EDTA-free Protease Inhibitor Cocktail, Roche Diagnostics, Mennheim, Germany) was then added to the recombinant protein solution at a rate of 1 tablet per 50 mL of solution. The RE15mR protein was precipitated from the solution by desalting with ammonium sulphate at 20–40% saturation at room temperature and separated from the solution by centrifugation. The resulting protein pool was dissolved in 20 mM Tris buffer containing 10 mM EDTA, pH 8.0. Ammonium sulphate was removed by dialysis of the recombinant protein suspension into 20 mM Tris buffer with 10 mM EDTA pH 8.0 overnight at 4 °C.

Protein was purified on a Macro-Prep High Q Media anionite resin column (Bio-Rad Laboratories, Hercules, CA, USA) in an FPLC system. A solution of dissolved, salted protein equilibrated with 20 mM Tris pH 8.0 calibration buffer was applied to the column. The recombinant hybrid protein RE15mR did not bind to the bed and was therefore eluted from the column with calibration buffer in the first fractions collected. To regenerate the column, the bed-bound protein was eluted with elution buffer consisting of 20 mM Tris pH 8.0 with 1 M NaCl. Throughout the chromatographic separation, a flow rate of 1–2 mL/min was used and fractions were collected according to a minimum absorbance reading of 0.05. The concentration of the eluted protein was checked by the Bradford and BCA method (Pierce BCA Protein Assay Kit, Thermo Fisher Scientific Inc., Rockford, IL, USA). The resulting protein was purified from bacterial endotoxins using Pierce High-Capacity Endotoxin Removal Resin columns (Thermo Fisher Scientific Inc., Rockford, IL, USA) according to the manufacturer’s instructions. The final isolate was dialyzed to ddH 20 for 24 h at 4 °C. The resulting protein was lyophilized and stored at 4 °C.

SDS-PAGE electrophoresis under denaturing conditions was used to control the purification process of the recombinant RE15mR structural protein. An example of electrophoretic separation (Coomassie Blue G-250 staining) is provided in the Supplementary Materials.

### 2.4. Amino Acid Sequence by Mass Spectrometry Analysis and Purity Confirmation in HPLC Analysis

Mass spectrometry experiments were performed at the Mass Spectrometry Laboratory (IBB PAS, Warsaw, Poland).

For the molecule mass analysis, the protein RE15mR was resuspended in 100 μL 0.1% TFA in H_2_O before mass measurement. Next, samples were analyzed using LC-MS system Waters Acquity, column Acquity UPLC Protein BEH C4, 300A, 1.7 μm, 1 mm × 50 mm coupled to SYNAPT-G2, capillary (kV) 2.5000, acquisition mass range 100.000–3000.000.

For the identification of amino acids sequence, the sample was resuspended in 100 μL 0.1% TFA in H_2_O. Next, the cysteines were reduced by 1 h incubation with 20 mM tris(2-carboxyethyl)phosphine at 37 °C followed by 10 min incubation at room temperature with 50 mM methyl methanethiosulfonate. Digestion was performed 3 h on immobilized pepsin (Thermo Scientific) at RT. Next, peptides were acidified with 0.1% formic acid. Samples were analyzed using LC-MS system composed of Evosep One (Evosep Biosystems, Odense, Denmark) coupled to an Orbitrap Exploris 480 mass spectrometer (Thermo Fisher Scientific) via Flex nanoESI ion source (Thermo Fisher Scientific). Samples were loaded onto disposable Evotips C18 trap columns (Evosep Biosystems) according to the manufacturer protocol with minor modifications. Chromatography was carried out at a flow rate 250 nL/min using the 88 min preformed gradient on EV1106 analytical column (Dr Maisch C18 AQ, 1.9 μm beads, 150 μm ID, 150 mm long, Evosep Biosystems).

Data were acquired in positive mode with a data-dependent method: MS1 resolution was set at 60,000 with a normalized AGC target 300%, auto maximum inject time and a scan range of 300 to 1600 *m*/*z*. For MS2, resolution was set at 15,000 with a standard normalized AGC target, auto maximum inject time, and top 40 precursors within an isolation window of 1.6 *m*/*z* considered for MS/MS analysis. Precursors were fragmented in HCD mode with normalized collision energy of 30%.

Raw data were pre-processed with the Mascot Distiller software v. 2.4.2.0, then obtained peptide masses and fragmentation spectra were matched to the *E. coli* database, cRAP, and user database, when study modifying sequences was added, using the Mascot search engine (Mascot Daemon v. 2.4.0, Mascot Server v. 2.4.1, and Matrix Science).

The purity analysis of the two substances was carried out by UHPLC on a Waters AQUITY instrument with a PDA eλ detector. To optimize conditions, the chromatogram was monitored at three wavelengths (λ), 200, 210, and 220 nm, with the best results obtained at 200 nm. A Waters SunFire C8 3.5 μm, 2.1 × 100 mm column was used for the analysis. The separation conditions were as follows: products were eluted with a binary phase layout of 0.1% TFA (trifluoroacetic acid) in water and 0.1% TFA in acetonitrile, at a flow rate of 0.5 mL/min and in a column temperature of 30 °C.

### 2.5. Application of the RE15mR Protein as a Component of Hydrogels and Bioinks for Bioprinting

#### 2.5.1. Preparation of Biomaterial

The materials tested can be divided into two main groups: (i) material based on methacrylated gelatin and (ii) material based on dECM. The hydrogel was prepared as a composition of two main ingredients: recombinant RE15mR protein and 10% (*w*/*v*) methacrylated gelatin (TINTBIONIC GELMA 80; Polbionica Ltd., Warsaw, Poland) in 1xPBS with 1.85 mg/mL LAP (phenyl-2,4,6 -lithium trimethylbenzoylphosphinate) (Polbionica Ltd., Warsaw, Poland) as a photoinitiator. The recombinant protein was used at four concentrations: 0.1, 0.5, 1.0, and 1.5 mg/mL. The reference sample was 10% GelMa with LAP without the addition of recombinant protein. The bioink was based on cell-free extracellular matrix dECM obtained from the pancreas, enriched with the recombinant RE15mR protein. In addition to dECM (Printiss^®^ dECM-PAN; Polbionica Ltd., Warsaw, Poland) (bioink (81.27 mg dECM/mL), the tested material contains methacrylated gelatin (TINTBIONIC GELMA 80; Polbionica Ltd., Poland) (37.15 mg/mL) and methacrylated hyaluronic acid (TINTBIONIC HAMA; Polbionica Ltd., Poland) (5.57 mg/mL) and LAP (2.32 mg/mL). The RE15mR protein was used in the experiment at two concentrations: 0.1 and 1.5 mg/mL. The reference sample was material without the addition of recombinant protein.

#### 2.5.2. Rheology

The study of the rheological properties of the developed material was performed using an Anton Paar MCR 72 rheometer. The measurement of the storage and loss module depending on temperature was performed under conditions of deformation of 30% (for hydrogel) and 5% (for bioink), frequency of 1 Hz and in the temperature range of 10–40 °C. The measurement of the modulus depending on the change in the set strain of 0.01–100% was performed at a temperature of 20 °C at a frequency of 1 Hz. The rotational measurement of the dynamic viscosity of hydrogels was performed at a constant temperature of 20 °C and a constant shear rate of 2 1/s. However, the rotational measurement of the dynamic viscosity of bioinks was carried out at a constant temperature of 25 °C and at a constant shear rate of 100 1/s. All tests performed for the tested materials were carried out using a PP25 spindle, with a gap of 1 mm.

#### 2.5.3. Printability

The ability of the material to be printed was assessed using three tests: a fusion test of fibers printed in the form of a template, a collapse test of a fiber printed on a three-dimensional platform, and an assessment of fiber continuity during continuous bioink printing in a volume of 3 mL. The tests were performed using a BIO X™ printer (Cellink, Gothenburg, Sweden). Material printing parameters were optimized by performing a series of extrusion tests under various temperature and pressure conditions. In order to perform the fiber merging test, two layers were printed one after the other using the tested material without cross-linking with a UV-Vis lamp between them. The prepared print follows a pattern in a 0–90° pattern, which reproduces the 2D effect and increases the distance between the fibers (FD). The distance between the fibers was in the range of 1–5 mm with 1 mm increments. The printing speed, needle diameter, and printing distance used in the test are 20 mm/s, 0.609 mm, and 0.8 mm, respectively. The print was cross-linked with an external UV-Vis lamp of 365 nm for 15 s with a power of 13 W/cm^2^. Based on the results, two parameters were determined, i.e., the percentage of diffusion rate (Dfr), which is the ratio of the difference between the theoretical and actual pore surfaces to the theoretical pore surface, and printability (Pr) as the ratio of the square of the pore circumference to the product of 16 and the actual pore surface.

The mid-span deflection of the suspended fiber was analyzed to determine the material’s collapse affinity. For this purpose, a special platform was designed and 3D printed, consisting of seven pillars spaced from each other by known distances of 1, 2, 3, 4, 5, 6 mm. The dimensions of the five posts placed inside the structure are 2 × 10 × 6 mm^3^, and the dimensions of the two edge posts are 5 × 10 × 6 mm^3^. A single fiber of the tested material was deposited on the platform using bioprinting technology. Printing was performed at a speed of 20 mm/s using a 21 G (0.609 mm) nozzle. The collapse area factor (Cf) was calculated as the percentage of the actual area after the suspended fiber was deflected from the theoretical area.

The continuity and smoothness of the fiber was assessed during continuous extrusion of 3 mL of bioink or hydrogel using optimal printing parameters in the 0/1 system, where when the fiber pulls it is 1 and when it breaks it is 0.

#### 2.5.4. Mechanical Properties Analysis

The mechanical properties of the printed constructs were evaluated using a static compression test. For this purpose, equipment was used consisting of the following elements: a computer with installed Axis FM v2_18 software, Pronterface, drivers for the force gauge and the tripod, a tripod with an electric drive and control, the Axis FB50 force gauge (maximum force 50 N) mounted on the tripod, and a printed compression head. In order to conduct the experiment, cylindrical samples with dimensions of diameter 10 mm and height 5 mm (100% filling, cross-linking with an external UV-Vis lamp after each layer) were designed and printed on a BIOX 3D printer (Cellink, Gothenburg, Sweden). All samples were initially loaded with a force from 0 to 0.05 N. The samples were compressed at a constant speed of 10 mm/min at room temperature until 80% deformation was obtained, with points collected every 0.025 s. Based on the results of the force-measurement time dependence, the strength was calculated mechanical test as the maximum stress (ratio of force to the surface of the printed sample) and Young’s modulus as the slope coefficient of the simple dependence of stress on sample deformation in the deformation range of 0.1–0.5. The agreed yield strength Re0.01 is the stress at which the sample is permanently deformed by 0.01% of its height.

#### 2.5.5. Analysis of Water Absorption

The water absorption capacity of the tested material was tested. For this purpose, 200 µL of biomaterial was poured onto the plates and cross-linked with light with a wavelength of 405 nm, power of 13 W/cm^2^, for 20 s in 3 repetitions. Ten mL of deionized water was added to the dishes and parafilmed. It was left at room temperature for 24 h. After 24 h, the water was removed and the dish was dried, weighed again, and then 10 mL of water was added again, parafilmed, and left for another 24 h at room temperature. Weighing and replacing deionized water was repeated after 48 and 72 h. The degree of water absorption (water content per mg of cross-linked material) can be calculated using the following formula:

(1) $$\mathrm{Water}\;\mathrm{absorption}\;[\%]=\frac{W_{N}-W_{M}}{W_{M}}\cdot 100\%$$

where

W_N_—The mass of the biomaterial after soaking at a given time point

W_M_—Mass of the biomaterial after pouring and gelling

### 2.6. Biological Characterization—Adhesion, Proliferation, and Viability/Cytotoxicity Tests

#### 2.6.1. Cell Culture

L929 cell line—mouse fibroblasts, adherent cells growing in monolayer, ATCC cat. no. CCL-1. Cells cultured in DMEM (Dulbecco’s Modified Eagle’s Medium) supplemented with 10% FBS, 4 mM L-glutamine, 4.5 g/L glucose, 1 mM sodium pyruvate, 1500 mg/L sodium carbonate, and 50 I.U./mL penicillin and 50 μg/mL streptomycin.

#### 2.6.2. Adhesion and Proliferation Tests for the RE15mR Protein as a Coating Agent

The experiments used the method of adventitial staining of cell cultures using AlamarBlue™ Cell Viability Reagent (Invitrogen, Eugene, OR, USA).

Adhesion and proliferation assays were performed on 96-well microplates designed for suspension culture (unmodified well surface). Lyophilized RE15mR protein was resuspended in water, applied to the plates at 1 and 5 μg/cm^3^ and the plates were dried overnight under sterile conditions. The control in this study was fibronectin applied to the coating at 1 μg/cm^3^. The negative control was the absence of any protein coating.

In the adhesion assay the following day, L929 cells were seeded into plates at 1 × 10^4^/well. Cells were incubated for 2, 4, and 24 h in dedicated culture medium. After the indicated time, dead or non-adherent cells were washed with sterile PBS buffer. For the proliferation assay, the number of L929 cells seeded in the plates was reduced (5 × 10^3^/well) while the culture times were increased (2, 24, and 48 h). The culture medium was then removed and the wells washed with PBS buffer to remove dead and non-adherent cells. In both assays, the culture was flooded with fresh medium and the cells were stained with AlamarBlue reagent for three hours, after which the fluorescence was measured at a light wavelength of 530 nm (excitation) and 590 nm (emission).

#### 2.6.3. Cytotoxicity of the RE15mR Protein in Relation to L929 Fibroblasts

Two methods, the classical MTT assay and calcein staining (LIVE/DEAD Viability/Cytotoxicity Kit for mammalian cells, Invitrogen, Eugene, OR, USA), were used to study the toxicity of the RE15mR protein.

The cytotoxicity of the RE15mR protein in solution against cells of the L-929 line (measurement of absorbance in the MTT assay) was tested according to ISO 10993-5:2009(E): *Biological evaluation of medical devices. Part 5:* In vitro *cytotoxicity test.* Cells were seeded at a density of 1 × 10^5^/mL or 5 × 10^4^/mL at 100 μL per well in 96-well plates, depending on the intended exposure time. To allow the fibroblasts to adhere to the bottom of the wells, they were cultured for 24 h under standard conditions (5% CO_2_ and 37 °C) in supplemented DMEM medium. The assay was performed using a direct method by adding a solution of purified reconstituted protein in medium to the culture. After controlling for confluence and population condition, 100 μL of a solution of protein in medium at concentrations of 1, 0.5, and 0.1 mg/mL was applied to the plates. The plate containing cells at a density of 1 × 10^5^/mL was incubated for 24 h, while the culture at the initial density of 5 × 10^4^/mL was exposed to RE15mR protein for 48 h. The cells were then incubated with MTT reagent solution for 2 h. All liquid above the cells was removed and the resulting formazan salt crystals were dissolved with DMSO. The amount of the resulting color product proportional to the number of viable cells was determined by measuring the absorbance at light wavelengths of 570 and 650 nm. According to the standard, viability of cells exposed to cytotoxic agent is expected to be at least 70% of that of untreated negative control cells.

In the second assay, purified RE15mR protein, after lyophilization, was used to coat 96-well plates intended for cell culture in suspension. An aqueous solution of RE15mR protein at 1 μg/cm^3^ was applied to the plates and dried. Fibronectin, also at 1 μg/cm^3^, was used as a positive control in the assay. The next day, L929 cells were seeded into the prepared plates at 2 × 10^4^/well, 1 × 10^4^/well, and 5 × 10^3^/well. Cells were incubated for 24, 48, and 72 h in dedicated culture medium. Cells were stained with calcein AM (1 μM) for 30 min and fluorescence was measured at light wavelengths of 485 nm (excitation) and 530 nm (emission).

#### 2.6.4. RE15mR Supplemented Biomaterial Cytotoxicity

An indirect MTT colorimetric assay was used to evaluate the proliferation of the L-929 cell line. For this purpose, extracts of fragmented biomaterials on the base of 10% GelMa (RE15mR tested concentrations 0.1, 0.2, 0.5, 1, and 1.5 mg/mL) were prepared by depositing them on inserts for 24 h in DMEM culture medium. Next, the extracts and positive control (CP, 0.1% Triton/DMEM) were added to the cells, which were seeded into 96-well plates at a density of 5 × 10^3^/well, 2.5 × 10^3^/well, and 1.25 × 10^3^/well and incubated at 37 °C, 5% CO₂ in DMEM medium for 24 h, 48 h, and 72 h, respectively. At the end of the incubation, the medium/extracts were removed and 1 mg/mL MTT was added. As before, an absorbance was measured at 570 and 650 nm. The viability of cells cultured with extracts was calculated in relation to untreated cell culture (CN, negative control).

#### 2.6.5. Viability Test for L929 Fibroblasts in 3D Test Bioprints

To test the viability of the cells in the bioink, test prints were prepared. Each print contained 25 μL of the mixture. L929 cells were resuspended in 10% GelMa and two variants of recombinant protein addition to the bioink were tested: high concentration (1 mg/mL) and low concentration (0.1 mg/mL). After 1 and 2 days of incubation in culture medium, the cells were stained with calcein AM (1 μM) and EthD-1 for 1 h and observed under the microscope for green and red fluorescence. The result was photographic documentation.

## 3. Results and Discussion

Although both resilin and elastin are known for properties such as high resilience, large strain, and low stiffness, as proteins they have very different physicochemical properties [17,40]. The arrangement of the resilin and elastin domains in the presented protein should allow distinct separation of the resilin hydrophilic fragment from the central part consisting of the elastin sequences and the tail of the C-terminal RGD repetitions. The presence of six resilin repeats at the N-terminus of the peptide chain was expected to increase the solubility of the designed protein in aqueous solutions [40]. We suspected that the designed protein, like the previously described artificial elastin derivatives [18], would be characterized by an inverse transition temperature and that the subsequent purification of RE15mRs would be possible according to the protocols proposed in the literature [41]. Unfortunately, centrifugation of the protein after heating did not lead to positive results, as too much of the total RE15zipR pool remained in the supernatant. Therefore, we used a different strategy, taking advantage of the thermostability of this protein, and heated the post-culture protein mixture to 90 °C in one step of the purification process. It was observed that while other proteins denatured and precipitated out of solution, the RE15mR protein remained in solution in a dissolved form. Additional lysine residues were positioned in the central part of the resilin and between the elastin sequences to ensure that the peptides would cross-link with the biomaterial components. Various chemical substances are known to enable cross-linking [42], but this molecule design assumed that the metacrylation of both RE15mR and other components of the biomaterial would be carried out by LAP [43] (cytocompatible photoinitiator type I)-mediated cross-linking using 405 nm light.

The designed protein molecule does not contain markers to facilitate the purification process, such as the [His]_6_-tag or other commercially available tags popular in scientific research and the pharmaceutical industry [41]. From the beginning of the project, it was assumed that the use of such a tag would enforce a downstream removal, for example by an enzymatic reaction, which is necessary to avoid due to the high cost of obtaining the final protein. Therefore, although the developed purification method requires a number of sequential steps, it is applicable to larger than typical amounts of protein in a laboratory scale. Results of purity analysis confirmed a high content of produced protein in samples obtained with optimized purification protocol (above 98% of RE15mR in tested batches). The amino acid sequence of the resulting protein was confirmed by peptide mapping after sample digestion with pepsin and analysis by mass spectrometry. The molecular mass of the protein produced was determined as 25.5 kDa using the mass spectrometry method, in accordance with the predicted theoretical value from the designed amino acid sequence.

**Figure 2:**
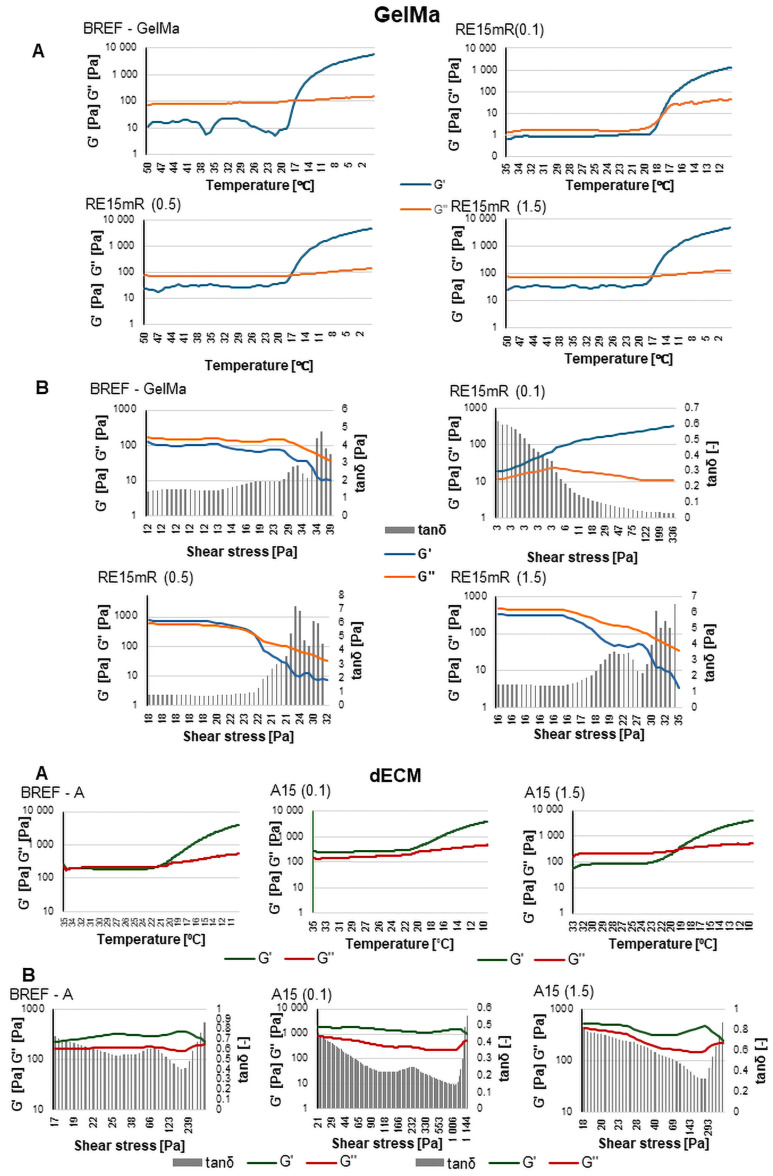
Assessment of rheological properties of materials for the use of GelMa and dECM recombinant RE15mR results, where (**A**) gelation point, (**B**) complex modulus.

The experiments carried out revealed that the resulting recombinant RE15mR protein is capable of gelling at concentrations above 200 mg/mL at 4 °C with 0.5 M NaCl. The characterized protein was tested for the possibility of using it as a hydrogel or bioink component with high utility in 3D bioprinting technology. Based on the results from rheological measurements, the sol-gel phase transition point, the dependence of the storage modulus and loss modulus on shear stress, and the average viscosity at a given temperature at a constant shear rate were identified. The use of recombinant protein in the hydrogel does not affect the temperature of the sol-gel phase transition point, which was 17 °C for both the reference sample and the tested material. However, changes in modulus values were identified under the influence of RE15mR protein concentration. The use of a high concentration of recombinant protein in the hydrogel had no effect on the relationship between the storage modulus and the shear stress; the value of the loss modulus was higher than the value of the storage modulus, which indicates that the viscous properties prevail over the elastic ones (Figure 2).

The use of a relatively low protein concentration leads to an initial increase in the storage modulus value compared to the loss modulus value for low shear stresses. The dynamic viscosity values in hydrogels containing the recombinant protein are RE15mR(0.1)—11.36 Pa·s, RE15mR(0.5)—13.90 Pa·s, and RE15mR(1.5)—17.33 Pa·s, respectively, while for the reference sample the viscosity is 24.11 Pa·s. The addition of the RE15mR recombinant protein to the hydrogel resulted in an increase in dynamic viscosity; the higher the protein concentration, the higher the viscosity of the biomaterial, which may be important for the use of this material in bioprinting technology [44,45]. The dynamic viscosity values in bioinks containing the recombinant protein are A15(0.1)—0.33 Pa·s and A15(1.5)—0.59 Pa·s, respectively, while for the reference sample the viscosity is 0.46 Pa·s. The use of RE15mR protein in a relatively low concentration of 0.1 mg/mL as an addition to the bioink containing dECM leads to a 30% decrease in the dynamic viscosity value, while the use of the protein in a high concentration of 1.5 mg/mL leads to an increase in viscosity by 30%. It has been reported that the viscosity of the bioink is a determining factor in the shape fidelity of the print; however, high viscosity does not necessarily ensure high mechanical strength or printing accuracy. An important element is the dependence of the storage modulus and loss on stress, which indicates the nature of the tested material. The use of the RE15mR protein at a relatively low concentration of 0.1 mg/mL causes a significant increase in the values of the complex module components compared to the reference bioink. This material has a G′ modulus higher than G″, which indicates greater elastic properties than viscous ones, which can also help with fiber stability and print resolution. 

**Figure 3:**
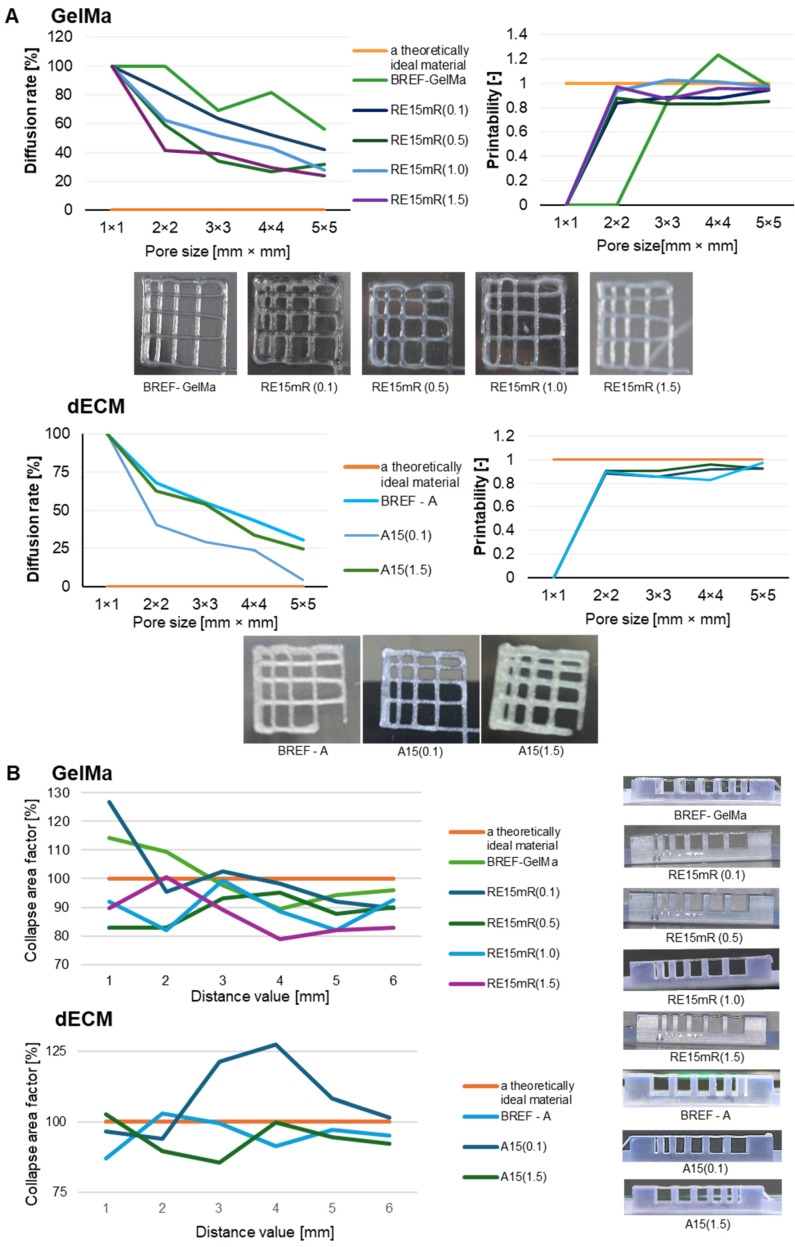
The printability of biomaterials based on GelMa and dECM containing the RE15mR recombinant protein, where (**A**) results of the fiber fusion test, (**B**) results of the fiber collapsing test.

There are a number of bioprinting techniques, such as material extrusion, direct energy deposition, powder bed fusion, sheet lamination, material spraying, binder spraying, and VAT photopolymerization [36]. Each of the above-mentioned techniques has a number of advantages and limitations mainly related to the characteristics of the materials used. Our research work included evaluating the printability of proprietary biomaterial compositions using an extrusion technique with commonly used tests such as fiber bonding, fiber collapse, and fiber continuity evaluation. The effects of the fiber fusion test as a measure of the material’s printability and resolution are presented on Figure 3. The results obtained for both the GelMa 10% bioink and the dECM bioink allow us to conclude that the diffusion rate decreases and the printability increases with the increase in the pore size of the printed pattern. The material containing the recombinant protein is characterized by high printing resolution, clearly better than the reference material without the addition of RE15mR. In the fiber collapse test as a measure of its stability, it was found that both tested variants of biomaterials containing the RE15mR recombinant protein are characterized by fiber continuity and stability. The collapse rate in both variants (even with such small supplementation as 0.1 mg/mL) was over 80% [46,47,48]. It appears that the addition of RE15mR has no effect on the continuity of the fiber and it promotes the retention of its shape in space. Figure 3 shows the test results demonstrating the continuity and smoothness of the fiber. The tested hydrogels containing GelMa are printable in the range of the following printing parameters, temperature, 21–23 °C and pressure 35–55 kPa, while bioinks containing dECM are printable in the range of temperatures 23–25 °C and pressure 35–45 kPa. It is worth mentioning that a biomaterial with high utility in cell bioprinting technology should meet several requirements, including the following: it should be efficiently printable under conditions close to physiological temperature, and the construct should be fixed under relatively mild conditions [49].

**Figure 4:**
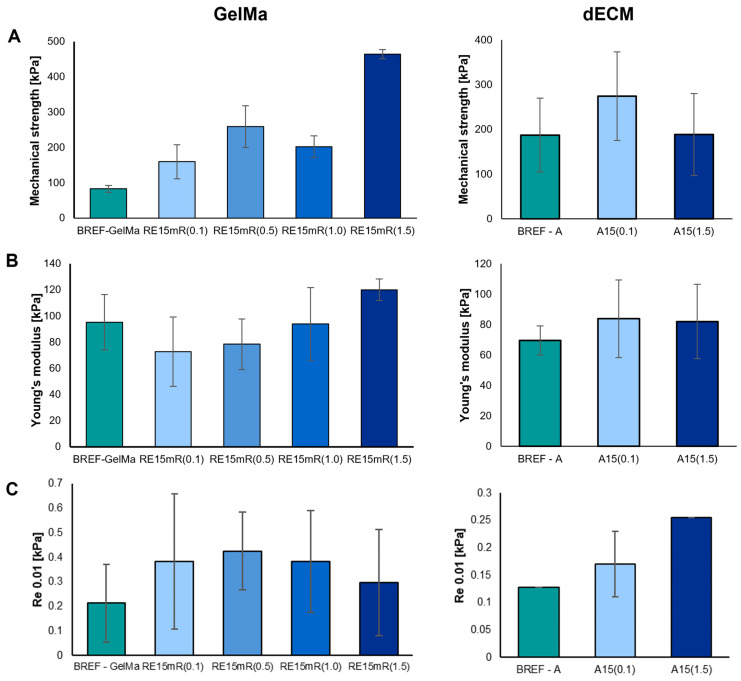
Mechanical parameters of bioinks based on 10%GelMa and dECM containing RE15mR protein: (**A**) mechanical strength, (**B**) Young’s modulus, (**C**) conventional yield strength.

The mechanical parameters of the printed constructs were determined—mechanical strength, Young’s modulus, and conventional yield strength. The measurement results are presented in Figure 4. The mechanical properties of a structure refer to its mechanical strength and degradation. It is worth noting that the materials of the construct after implantation degrade as the tissue regenerates. As a result, the mechanical properties of the constructs are dynamic, and the decrease in biomaterial strength caused by degradation is balanced by the increase in mechanical strength resulting from tissue regeneration. Ultimately, the mechanical properties of the construct should be similar to those of the tissue/organ repaired during regeneration [50,51,52,53,54]. The use of the recombinant RE15mR protein as an additive leads to significant changes in the mechanical parameters of printed constructs from both tested biomaterials. An increase in the concentration of the RE15mR protein in the hydrogel containing GelMa or dECM bioink leads to an increase in the value of the mechanical strength of the structure of the printed construct compared to the reference sample. The addition of the RE15mR protein in a relatively low concentration in the range of 0.1–0.5 mg/mL to the GelMa-based hydrogel leads to a decrease in the Young’s modulus of the printed construct compared to the reference sample. The use of recombinant protein in bioink containing dECM does not have a significant impact on the values of the following mechanical parameters: mechanical strength and Young’s modulus.

**Figure 5:**
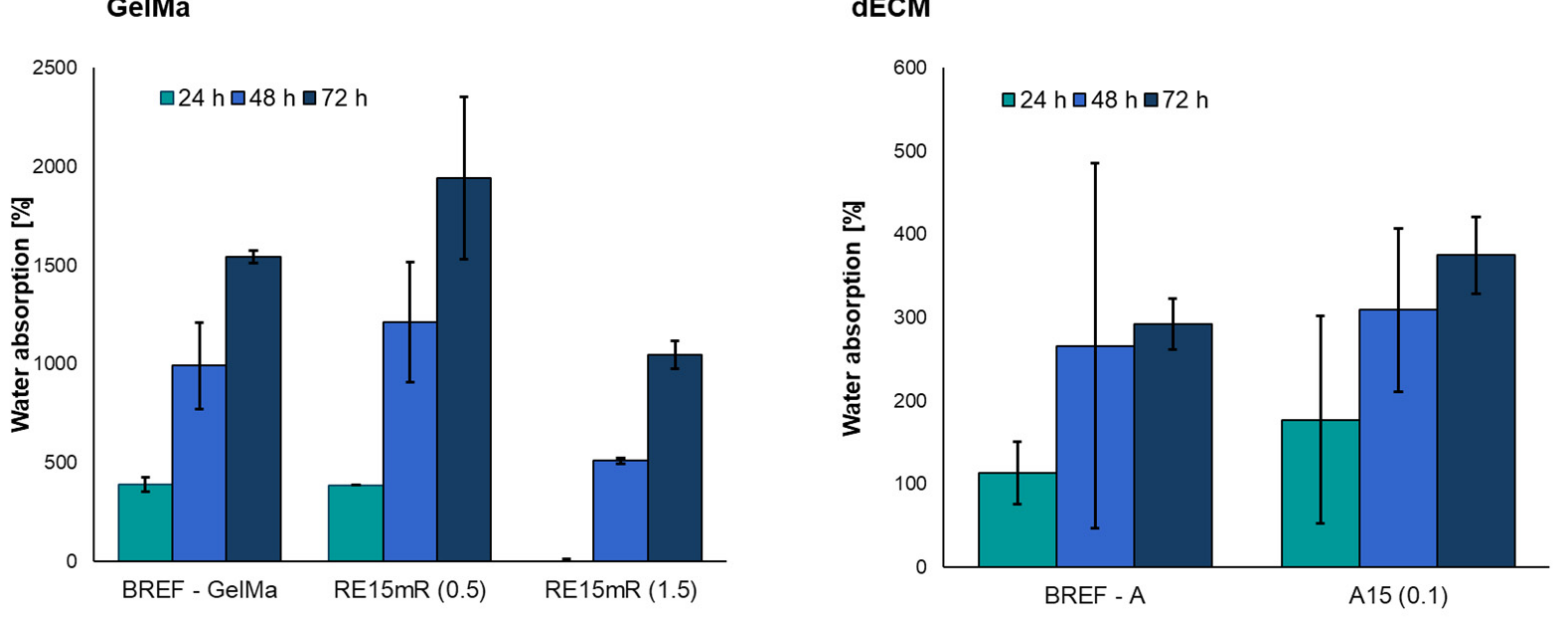
Water absorption of bioinks based on 10% GelMa and dECM containing RE15mR protein.

The use of a low concentration of recombinant RE15mR protein resulted in an increase in the degree of water absorption compared to the reference sample (Figure 5). However, a three-fold increase in protein concentration significantly reduced the degree of absorbability of the biomaterial compared to the reference sample.

The obtained results show that the addition of the recombinant RE15mR protein increases the degree of water absorption compared to the reference bioink A. The ability to absorb water by the construct is important in the case of, e.g., bone scaffolds, because it reflects the efficiency of the absorption of body fluids and the transport of nutrients to cells [55,56].

In order to develop a material with unique properties for printing, several important aspects must be taken into account—what print efficiency is important, which is closely related to the area of application of the printed construct. Therefore, the work involved the development of materials based on the GelMa hydrogel and dECM enriched with the RE15MR recombinant protein in order to improve their functional properties, i.e., biological and physicochemical properties. The addition of recombinant RE15mR protein has a significant impact on the rheological parameters of the material. The increase in concentration leads to an increase in dynamic viscosity and storage modulus, which makes the material have stronger elastic properties than viscous ones, which guarantees better printing resolution. All tested materials are printable under relatively mild printing conditions with the desired efficiency. The increase in protein content leads to improved mechanical parameters, i.e., an increase in the mechanical strength and elasticity of the printed construct, which is important for the use of this material for printing structures, e.g., models with a vascular system, requiring significant resistance to shear stresses. Moreover, it has been shown that the material containing the recombinant protein slightly increases the degree of water absorption compared to the reference material.

The biocompatibility of biomaterial inks in 3D bioprinting is mainly reflected in the non-toxic effect of maintaining or enhancing cell proliferation [57]. Biomaterial ink should be safe to use in the host organism or in endogenous tissue in order to avoid toxic effects or an immunological reaction in the organism. It is expected that the implant material should mimic biological functions and improve the living microenvironment. That is why preliminary biological tests with purified protein alone and as additives to bioink were made. AlamarBlue staining was used for adhesion and proliferation studies. The active ingredient in this method is resazurin, a non-toxic blue compound which, when entering living cells in a reducing environment, is converted to red, highly fluorescent resorufin. This test is used to determine the number of living cells that are metabolically active.

**Figure 6:**
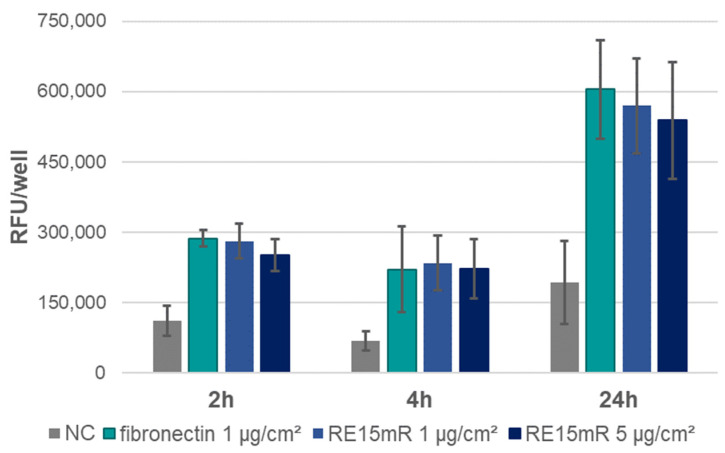
Adhesion of L929 cells—fluorescence assay (AlamarBlue staining) in cell culture on microtiter plates coated with RE15mR protein, with fibronectin as a positive control. The graph shows the mean value of the results obtained in the three experiments.

**Figure 7:**
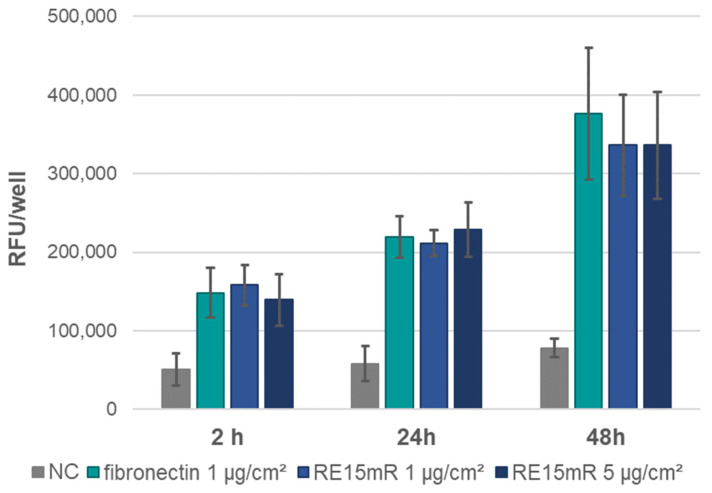
L929 cell proliferation assay—fluorescence measurement in AlamarBlue staining of cells on plates coated with RE15mR protein compared to fibronectin. The mean results of the three experiments are shown in the graph.

In the adhesion assay, the increase in fluorescence during incubation (Figure 6) indicates cell adhesion to the plastic used, both in the positive control (fibronectin) and in the wells coated with the test protein (RE15mR at two concentrations). It was observed that the RE15mR protein promoted cell adhesion of the cell line used in this study to a comparable level to fibronectin, a commercially available protein used for the coating of surfaces intended for eukaryotic cell culture. In subsequent experiments, measurement of fluorescence in culture at 48 h versus 2 and 24 h, taking into account the division time for the L929 cell line (Figure 7), indicates active cell proliferation in both the positive control and test protein coated wells. Therefore, the cell proliferation rate of the cell line used in this study is not affected by the RE15mR protein.

**Figure 8:**
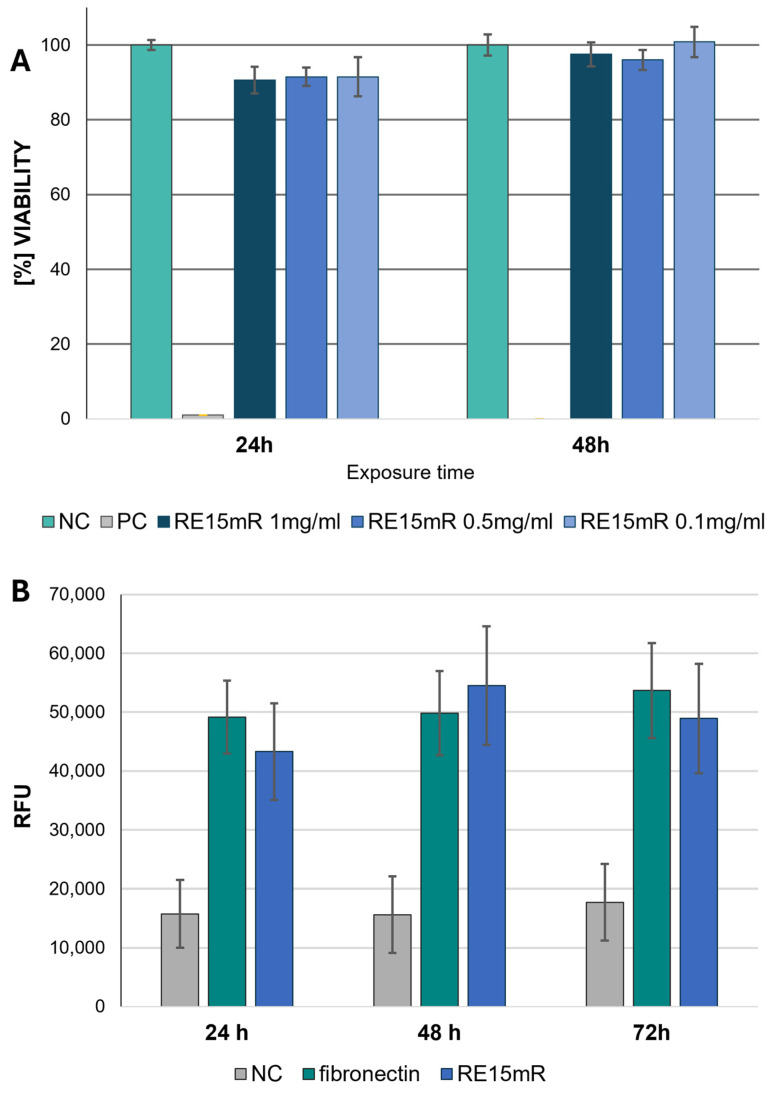
Cytotoxicity of RE15mR protein against L929 cells. Direct test MTT results (**A**); green fluorescence measurement by calcein staining (**B**) using plates coated with 1 μg/cm^3^ of RE15mR protein compared to fibronectin.

Based on the results obtained in the MTT assay, RE15mR protein in solution was not cytotoxic to the L929 fibroblast line in the concentration range tested (0.1–1 mg/mL) at 24 and 48 h exposure (Figure 8A). These observations support the results of the cell viability is fluorescence staining with a pair of reagents (to indicate live and dead cells), calcein and EthD-1. Non-fluorescent acetylmethoxy calcein (AM calcein) freely penetrates cell membranes and is enzymatically converted in the cell to intensely fluorescent green calcein, making it suitable for live cell imaging. The ethidium homodimer EthD-1 enters cells through damaged membranes and, when bound to nucleic acids, produces an increase in bright red fluorescence, allowing the detection of dead cells in the population. The use of calcein AM/EthD-1 staining allows the qualitative and quantitative assessment of the proportion of living and dead cells in the population, and also the preservation of their shape and position. Unlike the MTT method (the reference method with formazan salt staining), it does not require dissociation of the cells for the measurement and allows microscopic observation in situ. Therefore, the results obtained from the MTT test were compared with calcein and EthD-1 staining. It can be concluded that the RE15mR protein, when used to coat the culture plastic, is not cytotoxic to L929 cells to the same extent as fibronectin, which is commonly used for this purpose, regardless of culture time, based on measurements of the increase in green fluorescence during incubation (Figure 8B).

**Figure 9:**
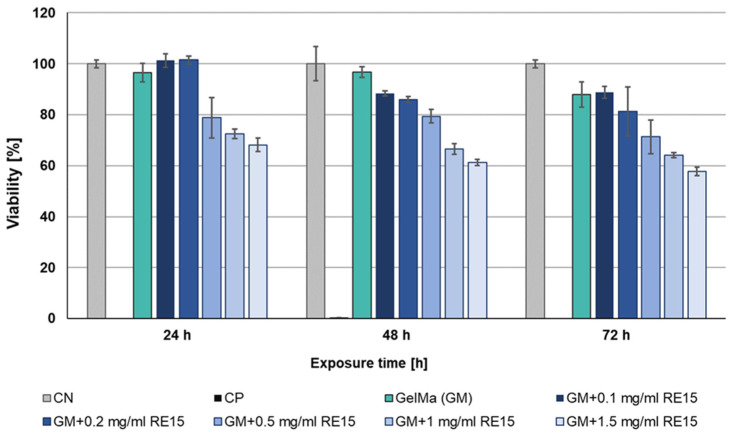
Viability of L929 cell line in an indirect MTT test with biomaterial (10% GelMa) supplemented with various concentrations of RE15mR protein.

The high result of measuring cell viability in the presence of the RE15mR protein inspired studies of the cytotoxicity of biomaterials with its addition. The MTT assay was performed again, this time indirect, performed at 24, 48, and 72 h after the application of extracts derived from biomaterials. Based on the assay results, the cytotoxic effect of the extract variants with ≤0.5 mg/mL of recombinant protein on L929 cell line was not observed. The lack of cytotoxicity was observed for all tested biomaterials (0.1–1 mg/mL of supplement) after 24 h exposure with the exception of the highest dose. During the next days of incubation, cell viability gradually begins to slightly decrease. But still, for biomaterials with additive 0.1, 0.2, and 0.5 mg/mL, viability is above 70%, which determines the lack of cytotoxicity for the material tested (Figure 9). In addition, the analysis of the morphology variations of the L929 cell line using microscopic imaging was also performed. Based on the microscopic images, there were no deviations in the morphology of the cells when they were incubated during time with extracts derived from biomaterials. In conclusion, in the tested biomaterials, a RE15mR protein content of less than ≤0.5 mg/mL is not cytotoxic for the fibroblasts of the reference cell line.

**Figure 10:**
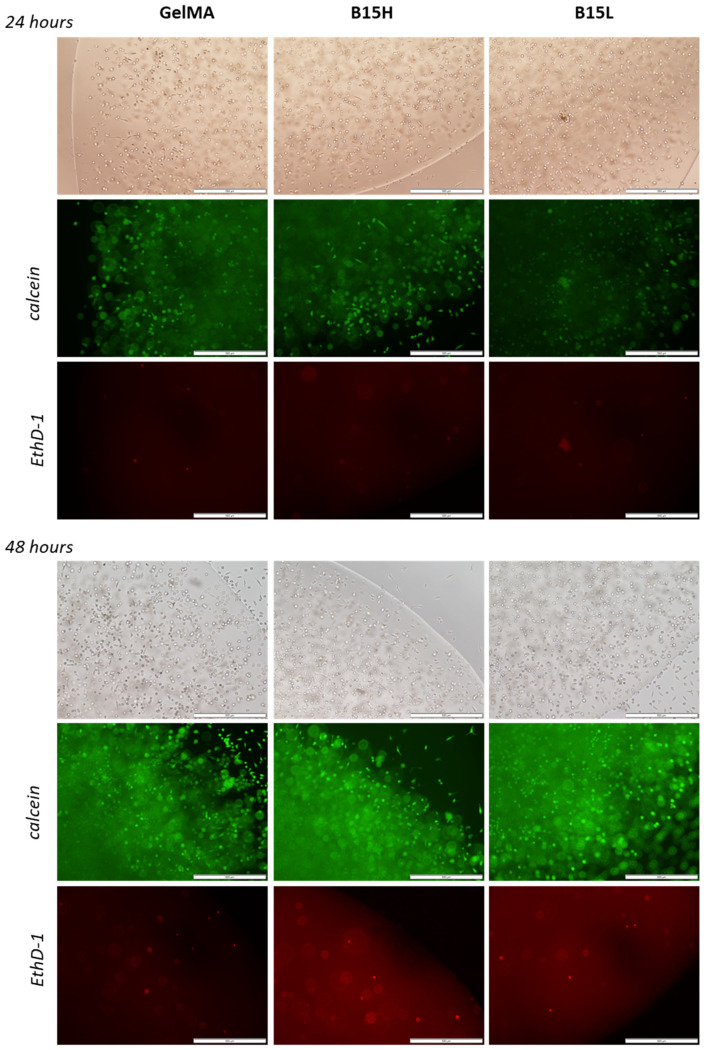
Images of 3D prints of L929 cells stained with calcein (live cells, green) and EthD-1 (dead cells, red) as observed by microscopy (4-fold objective magnification) under visible light and fluorescence after 24 and 48 h of culture. From left: control samples—bioink with 10% methacrylated gelatine, B15H—bioink supplemented with 1 mg/mL of RE15mR, B15L—bioink with 0.1 mg/mL of RE15mR protein. The scale bar is 1 mm.

Calcein and EthD-1 staining of L929 cells in the 3D printed samples (images combined in the panel in Figure 10) confirmed the high viability of cells grown in the microenvironment containing the added recombinant RE15mR protein. The number of viable cells was not adversely affected by the addition of even 1 mg RE15mR/mL bioink, nor was the mortality of the population increased. The attached images show that the viability level of the cell population did not decrease during the two-day culture and that the biomaterial used for the prints promoted the proliferation of the cells embedded within. Cells visible outside of the print suggest that the biomaterial used does not restrict cell migration. The results confirm previous observations that the RGD sequences identified in the fibronectin molecule actively promote cell adhesion and proliferation of various cell types [32,33,34,35], including fibroblasts. Therefore, their incorporation into an engineered protein or peptide can alter the properties of the surrounding biomaterial, creating a microenvironment favorable to cell colonization. This observation is consistent with previous reports of the use of elastin or its derivatives, e.g., recombinant tropoelastin, in 3D printing biomaterials [4,58], which, in combination with other structural proteins like collagen [59] or functional domains like the RGD sequence, created favorable conditions for cell settlement and growth.

The right composition of biomimetic components in printed constructs can have a significant impact on cell adhesion, migration, proliferation, differentiation, and other biological functions. Although the basis of many bioinks remains native dECM, as this is the natural environment for cell growth in vivo, bioinks today are differentiated by the addition of components that alter or enhance selected features of biological activity [57]. In terms of both mechanical properties and biological activity, the presented recombinant RE15mR protein appears to be an interesting component alternative to native proteins used in 3D bioprinting.

## 4. Conclusions

This work describes the design, production, and characterization of a genetically modified low molecular weight chimeric protein. A protein inspired by repeated sequences of resin and elastin is a completely new component in tissue engineering.

The combination of the functional properties of these two proteins with the addition of numerous repeats of the RGD sequence resulted in valuable properties of this recombinant protein. Its properties can be used in many aspects of regenerative medicine and tissue engineering. First of all, protein can be safely added as a component for 3D bioprinting of tissues and organs using the extrusion method. Its mechanical, physicochemical, and biological properties suggest that it cannot only modify the features of the print in terms of plasticity or strength, but also help create a favorable environment for the colonization of printed constructs with cells. The use of the RE15mR protein as a bioink component improves the rheological properties of the biomaterial and affects the mechanical parameters of the bioink and water absorption. Preliminary in vitro biological tests demonstrated the potential of the RE15mR protein as a matrix component in cell culture and as a hydrogel component. However, going a step further, it can also be used as a coating matrix, e.g., for hard biomaterials. An increasingly common trend in modern tissue engineering is to combine artificial and natural materials. In this aspect, the produced protein may be a revolutionary discovery for this group of materials. Covering the biopolymer with our protein can significantly affect the colonization of materials with varying degrees of hardness with living cells, not necessarily natural ones.

We hope that the use of RE15mR as a biotechnological component in tissue and organoid engineering will be a step towards the future production of complex, multifunctional tissues and organs for use in regenerative medicine.

## 5. Patents

Patent application resulting from the work reported in this manuscript: PCT/PL2024/050017.

## Data Availability

Data are contained within the article and Supplementary Materials.

## Supplementary Materials

The following supporting information can be downloaded at: https://www.mdpi.com/article/10.3390/nano14090749/s1, Figure S1: Map of the expression vector RE15mR_pET-11a containing a gene coding hybrid protein RE15mR; Figure S2: Amino acid sequence of the recombinant hybrid protein RE15mR; Figure S3: Electrophoretic separation’s example of samples taken during the RE15mR purification process.

## References

[1] Mu X., Agostinacchio F., Xiang N., Pei Y., Khan Y., Guo C., Cebe P., Motta A., Kaplan D.L. (2021). Recent Advances in 3D Printing with Protein-Based Inks. Prog. Polym. Sci..

[2] Wang X., Wang Q., Xu C. (2020). Nanocellulose-Based Inks for 3d Bioprinting: Key Aspects in Research Development and Challenging Perspectives in Applications—A Mini Review. Bioengineering.

[3] Renner J.N., Cherry K.M., Su R.S.C., Liu J.C. (2012). Characterization of Resilin-Based Materials for Tissue Engineering Applications. Biomacromolecules.

[4] Lee S., Sani E.S., Spencer A.R., Guan Y., Weiss A.S., Annabi N. (2020). Human-Recombinant-Elastin-Based Bioinks for 3D Bioprinting of Vascularized Soft Tissues. Adv. Mater..

[5] Wu Y., Lin Z.Y., Wenger A.C., Tam K.C., Tang X. (2018). 3D Bioprinting of Liver-Mimetic Construct with Alginate/Cellulose Nanocrystal Hybrid Bioink. Bioprinting.

[6] Khan F., Tanaka M. (2018). Designing Smart Biomaterials for Tissue Engineering. Int. J. Mol. Sci..

[7] Klak M., Bryniarski T., Kowalska P., Gomolka M., Tymicki G., Kosowska K., Cywoniuk P., Dobrzanski T., Turowski P., Wszola M. (2020). Novel Strategies in Artificial Organ Development: What Is the Future of Medicine?. Micromachines.

[8] Sengupta D., Heilshorn S.C. (2010). Protein-Engineered Biomaterials: Highly Tunable Tissue Engineering Scaffolds. Tissue Eng. Part. B Rev..

[9] White L.J., Taylor A.J., Faulk D.M., Keane T.J., Saldin L.T., Reing J.E., Swinehart I.T., Turner N.J., Ratner B.D., Badylak S.F. (2017). The Impact of Detergents on the Tissue Decellularization Process: A ToF-SIMS Study. Acta Biomater..

[10] Zhang C.Y., Fu C.P., Li X.Y., Lu X.C., Hu L.G., Kankala R.K., Wang S.B., Chen A.Z. (2022). Three-Dimensional Bioprinting of Decellularized Extracellular Matrix-Based Bioinks for Tissue Engineering. Molecules.

[11] Kabirian F., Mozafari M. (2020). Decellularized ECM-Derived Bioinks: Prospects for the Future. Methods.

[12] Syed O., Walters N.J., Day R.M., Kim H.W., Knowles J.C. (2014). Evaluation of Decellularization Protocols for Production of Tubular Small Intestine Submucosa Scaffolds for Use in Oesophageal Tissue Engineering. Acta Biomater..

[13] Fernández-Pérez J., Ahearne M. (2019). The Impact of Decellularization Methods on Extracellular Matrix Derived Hydrogels. Sci. Rep..

[14] Rieder E., Kasimir M.T., Silberhumer G., Seebacher G., Wolner E., Simon P., Weigel G. (2004). Decellularization Protocols of Porcine Heart Valves Differ Importantly in Efficiency of Cell Removal and Susceptibility of the Matrix to Recellularization with Human Vascular Cells. J. Thorac. Cardiovasc. Surg..

[15] Singh G., Satpathi S., Gopala Reddy B.V., Singh M.K., Sarangi S., Behera P.K., Nayak B. (2023). Impact of Various Detergent-Based Immersion and Perfusion Decellularization Strategies on the Novel Caprine Pancreas Derived Extracellular Matrix Scaffold. Front. Bioeng. Biotechnol..

[16] Cebotari S., Tudorache I., Jaekel T., Hilfiker A., Dorfman S., Ternes W., Haverich A., Lichtenberg A. (2010). Detergent Decellularization of Heart Valves for Tissue Engineering: Toxicological Effects of Residual Detergents on Human Endothelial Cells. Artif. Organs.

[17] Bracalello A., Santopietro V., Vassalli M., Marletta G., Del Gaudio R., Bochicchio B., Pepe A. (2011). Design and Production of a Chimeric Resilin-, Elastin-, and Collagen-like Engineered Polypeptide. Biomacromolecules.

[18] Bracalello A., Secchi V., Mastrantonio R., Pepe A., Persichini T., Iucci G., Bochicchio B., Battocchio C. (2019). Fibrillar Self-Assembly of a Chimeric Elastin-Resilin Inspired Engineered Polypeptide. Nanomaterials.

[19] Bochicchio B., Bracalello A., Pepe A. (2016). Characterization of a Crosslinked Elastomeric-Protein Inspired Polypeptide. Chirality.

[20] Aghaei-Ghareh-Bolagh B., Mithieux S.M., Weiss A.S. (2016). Elastic Proteins and Elastomeric Protein Alloys. Curr. Opin. Biotechnol..

[21] Li L., Kiick K.L. (2013). Resilin-Based Materials for Biomedical Applications. ACS Macro Lett..

[22] Rodgers U.R., Weiss A.S. (2005). Cellular Interactions with Elastin. Pathol. Biol..

[23] Weis-Fogh T. (1960). A Rubber-Like Protein in Insect Cuticle. J. Exp. Biol..

[24] Miao M., Bellingham C.M., Stahl R.J., Sitarz E.E., Lane C.J., Keeley F.W. (2003). Sequence and Structure Determinants for the Self-Aggregation of Recombinant Polypeptides Modeled after Human Elastin. J. Biol. Chem..

[25] Weitzhandler I., Dzuricky M., Hoffmann I., Garcia Quiroz F., Gradzielski M., Chilkoti A. (2017). Micellar Self-Assembly of Recombinant Resilin-/Elastin-Like Block Copolypeptides. Biomacromolecules.

[26] Elvin C.M., Carr A.G., Huson M.G., Maxwell J.M., Pearson R.D., Vuocolo T., Liyou N.E., Wong D.C.C., Merritt D.J., Dixon N.E. (2005). Synthesis and Properties of Crosslinked Recombinant Pro-Resilin. Nature.

[27] Simnick A.J., Lim D.W., Chow D., Chilkoti A. (2007). Biomedical and Biotechnological Applications of Elastin-like Polypeptides. Polym. Rev..

[28] Salinas-Fernández S., Santos M., Alonso M., Quintanilla L., Rodríguez-Cabello J.C. (2020). Genetically Engineered Elastin-like Recombinamers with Sequence-Based Molecular Stabilization as Advanced Bioinks for 3D Bioprinting. Appl. Mater. Today.

[29] Lutolf M.P., Lauer-Fields J.L., Schmoekel H.G., Metters A.T., Weber F.E., Fields G.B., Hubbell J.A. (2003). Synthetic Matrix Metalloproteinase-Sensitive Hydrogels for the Conduction of Tissue Regeneration: Engineering Cell-Invasion Characteristics. Proc. Natl. Acad. Sci. USA.

[30] Patterson J., Hubbell J.A. (2011). SPARC-Derived Protease Substrates to Enhance the Plasmin Sensitivity of Molecularly Engineered PEG Hydrogels. Biomaterials.

[31] Van Hove A.H., Antonienko E., Burke K., Brown E., Benoit D.S.W. (2015). Temporally Tunable, Enzymatically Responsive Delivery of Proangiogenic Peptides from Poly(Ethylene Glycol) Hydrogels. Adv. Healthc. Mater..

[32] Liu Q., Zheng S., Ye K., He J., Shen Y., Cui S., Huang J., Gu Y., Ding J. (2020). Cell Migration Regulated by RGD Nanospacing and Enhanced under Moderate Cell Adhesion on Biomaterials. Biomaterials.

[33] He J., Liu Q., Zheng S., Shen R., Wang X., Gao J., Wang Q., Huang J., Ding J. (2021). Enlargement, Reduction, and Even Reversal of Relative Migration Speeds of Endothelial and Smooth Muscle Cells on Biomaterials Simply by Adjusting RGD Nanospacing. ACS Appl. Mater. Interfaces.

[34] Sang Jeon H., Sil Lee J., Hur W. (2020). Enzymatic Conjugation of Rgd Peptides on the Surface of Fibroin Microspheres. Appl. Chem. Eng..

[35] Lateef S.S., Boateng S., Hartman T.J., Crot C.A., Russell B., Hanley L. (2002). GRGDSP Peptide-Bound Silicone Membranes Withstand Mechanical Flexing In Vitro and Display Enhanced Fibroblast Adhesion. Biomaterials.

[36] Ahmad M.N., Ishak M.R., Mohammad Taha M., Mustapha F., Leman Z. (2023). A Review of Natural Fiber-Based Filaments for 3D Printing: Filament Fabrication and Characterization. Materials.

[37] Paxton N., Smolan W., Böck T., Melchels F., Groll J., Jungst T. (2017). Proposal to Assess Printability of Bioinks for Extrusion-Based Bioprinting and Evaluation of Rheological Properties Governing Bioprintability. Biofabrication.

[38] Ng W.L., Huang X., Shkolnikov V., Suntornnond R., Yeong W.Y. (2023). Polyvinylpyrrolidone-Based Bioink: Influence of Bioink Properties on Printing Performance and Cell Proliferation during Inkjet-Based Bioprinting. Biodes Manuf..

[39] Levato R., Dudaryeva O., Garciamendez-Mijares C.E., Kirkpatrick B.E., Rizzo R., Schimelman J., Anseth K.S., Chen S., Zenobi-Wong M., Zhang Y.S. (2023). Light-Based Vat-Polymerization Bioprinting. Nat. Rev. Methods Primers.

[40] Charati M.B., Ifkovits J.L., Burdick J.A., Linhardt J.G., Kiick K.L. (2009). Hydrophilic Elastomeric Biomaterials Based on Resilin-like Polypeptides. Soft Matter.

[41] Yeboah A., Cohen R.I., Rabolli C., Yarmush M.L., Berthiaume F. (2016). Elastin-like Polypeptides: A Strategic Fusion Partner for Biologics. Biotechnol. Bioeng..

[42] Raman R., Bashir R. (2015). Stereolithographic 3D Bioprinting for Biomedical Applications. Essentials of 3D Biofabrication and Translation.

[43] Nguyen A.K., Goering P.L., Elespuru R.K., Das S.S., Narayan R.J. (2020). The Photoinitiator Lithium Phenyl (2,4,6-Trimethylbenzoyl) Phosphinate with Exposure to 405 nm Light Is Cytotoxic to Mammalian Cells but Not Mutagenic in Bacterial Reverse Mutation Assays. Polymers.

[44] Habib A., Sarah R., Tuladhar S., Khoda B., Limon S.M. (2024). Modulating Rheological Characteristics of Bio-Ink with Component Weight and Shear Rate for Enhanced Bioprinted Scaffold Fidelity. Bioprinting.

[45] Sánchez-Sánchez R., Rodríguez-Rego J.M., Macías-García A., Mendoza-Cerezo L., Díaz-Parralejo A. (2023). Relationship between Shear-Thinning Rheological Properties of Bioinks and Bioprinting Parameters. Int. J. Bioprint.

[46] Naghieh S., Chen X. (2021). Printability—A Key Issue in Extrusion-Based Bioprinting. J. Pharm. Anal..

[47] Habib A., Sathish V., Mallik S., Khoda B. (2018). 3D Printability of Alginate-Carboxymethyl Cellulose Hydrogel. Materials.

[48] Sarker M., Izadifar M., Schreyer D., Chen X. (2018). Influence of Ionic Crosslinkers (Ca^2+^/Ba^2+^/Zn^2+^) on the Mechanical and Biological Properties of 3D Bioplotted Hydrogel Scaffolds. J. Biomater. Sci. Polym. Ed..

[49] Raees S., Ullah F., Javed F., Akil H.M., Jadoon Khan M., Safdar M., Din I.U., Alotaibi M.A., Alharthi A.I., Bakht M.A. (2023). Classification, Processing, and Applications of Bioink and 3D Bioprinting: A Detailed Review. Int. J. Biol. Macromol..

[50] Chen X.B., Fazel Anvari-Yazdi A., Duan X., Zimmerling A., Gharraei R., Sharma N.K., Sweilem S., Ning L. (2023). Biomaterials/Bioinks and Extrusion Bioprinting. Bioact. Mater..

[51] Ahmadi Soufivand A., Faber J., Hinrichsen J., Budday S. (2023). Multilayer 3D Bioprinting and Complex Mechanical Properties of Alginate-Gelatin Mesostructures. Sci. Rep..

[52] Ding Y.W., Zhang X.W., Mi C.H., Qi X.Y., Zhou J., Wei D.X. (2023). Recent Advances in Hyaluronic Acid-Based Hydrogels for 3D Bioprinting in Tissue Engineering Applications. Smart Mater. Med..

[53] Yang J., He H., Li D., Zhang Q., Xu L., Ruan C. (2023). Advanced Strategies in the Application of Gelatin-Based Bioink for Extrusion Bioprinting. Biodes Manuf..

[54] Kannayiram G., Sendilvelan S., Priya R.M. (2023). Importance of Nanocomposites in 3D Bioprinting: An Overview. Bioprinting.

[55] Liu J., Li L., Suo H., Yan M., Yin J., Fu J. (2019). 3D Printing of Biomimetic Multi-Layered GelMA/NHA Scaffold for Osteochondral Defect Repair. Mater. Des..

[56] Shuai C., Yang W., Feng P., Peng S., Pan H. (2021). Accelerated Degradation of HAP/PLLA Bone Scaffold by PGA Blending Facilitates Bioactivity and Osteoconductivity. Bioact. Mater..

[57] Li X., Zheng F., Wang X., Geng X., Zhao S., Liu H., Dou D., Leng Y., Wang L., Fan Y. (2022). Biomaterial Inks for Extrusion-Based 3D Bioprinting: Property, Classification, Modification, and Selection. Int. J. Bioprint.

[58] Dai M., Belaïdi J.P., Fleury G., Garanger E., Rielland M., Schultze X., Lecommandoux S. (2021). Elastin-like Polypeptide-Based Bioink: A Promising Alternative for 3D Bioprinting. Biomacromolecules.

[59] Tytgat L., Dobos A., Markovic M., Van Damme L., Van Hoorick J., Bray F., Thienpont H., Ottevaere H., Dubruel P., Ovsianikov A. (2020). High-Resolution 3D Bioprinting of Photo-Cross-Linkable Recombinant Collagen to Serve Tissue Engineering Applications. Biomacromolecules.

