# Supplementary material for: Characterization of a Chimeric Resilin-Elastin Structural Protein Dedicated to 3D Bioprinting as a Bioink Component
Source: Nanomaterials (Basel). 2024 Apr 25;14(9):749. doi: 10.3390/nano14090749 (PMC11085090; doi:10.3390/nano14090749)
Supplement: Supplementary file 1 [file nanomaterials-14-00749-s001.zip › nanomaterials-2962338-supplementary.pdf]

SUPPLEMENTARY MATERIAL

Fig. S1: Map of the expression vector RE15mR_pET-11a containing a gene coding hybrid protein RE15mR.

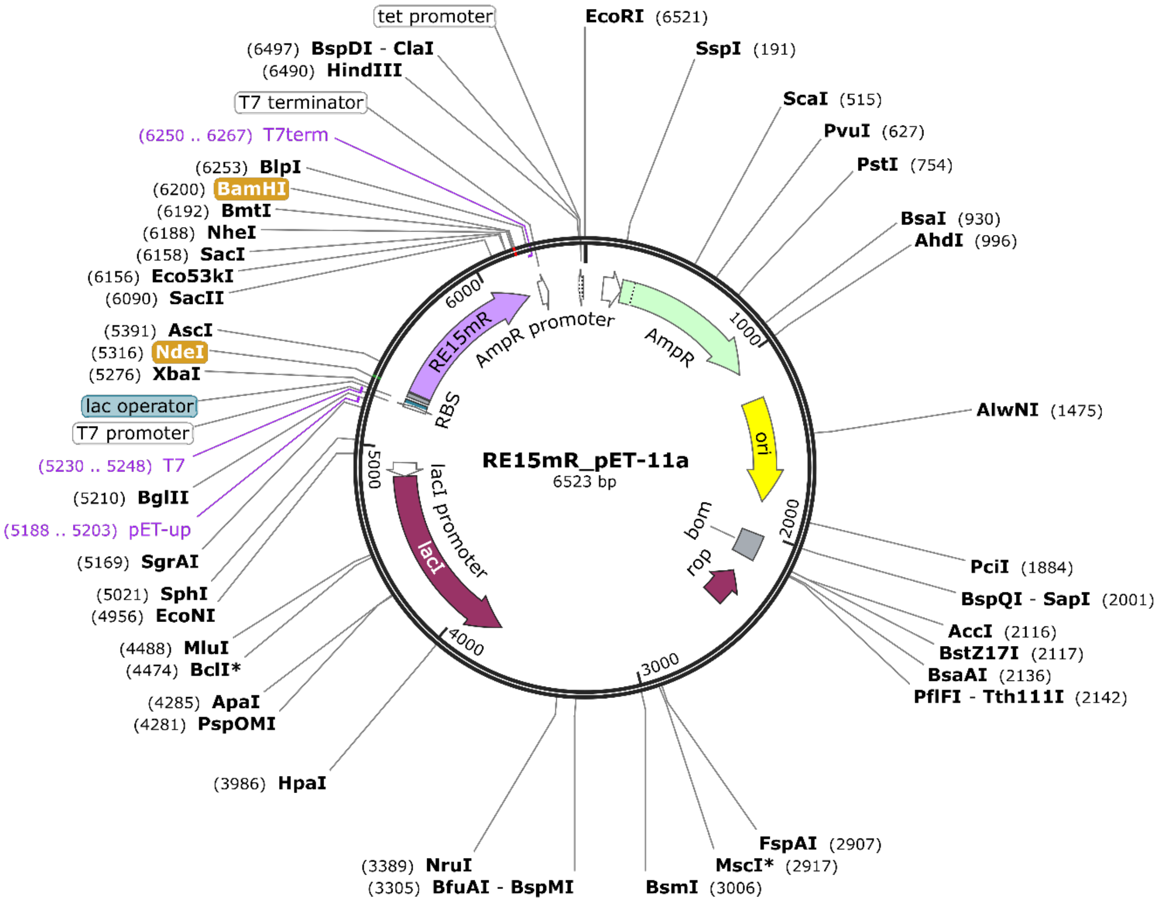

Fig. S2:Amino acid sequence of the recombinant hybrid protein RE15mR.

MGGRPSDSYGAPGGGNGGRPSDSYGAPGGGNGGRPSDSYGAPGGGNGGKGGKGGK

GGGGRPSDSYGAPGGGNGGRPSDSYGAPGGGNGGRPSDSYGAPGGGNGPQGIWGQ

GVPGAGVPGAGVPGAGKVPGIGVPGIGVPGIGVPGIGKVPGAGVPGAGVPGAGKVPG

IGVPGIGVPGIGVPGIGKVPGAGVPGAGVPGAGGPQGIWGQGAVTGRGDSPASSAVTG

RGDSPASSAVTGRGDSPASSAVTGRGDSPASSAVTGRGDSPASSAVTGRGDSPASSAVT GRGDSPASS

Fig. S3: Electrophoretic separation's example of samples taken during the RE15mR purification process (SDS-page, 15% polyacrylamide gel)

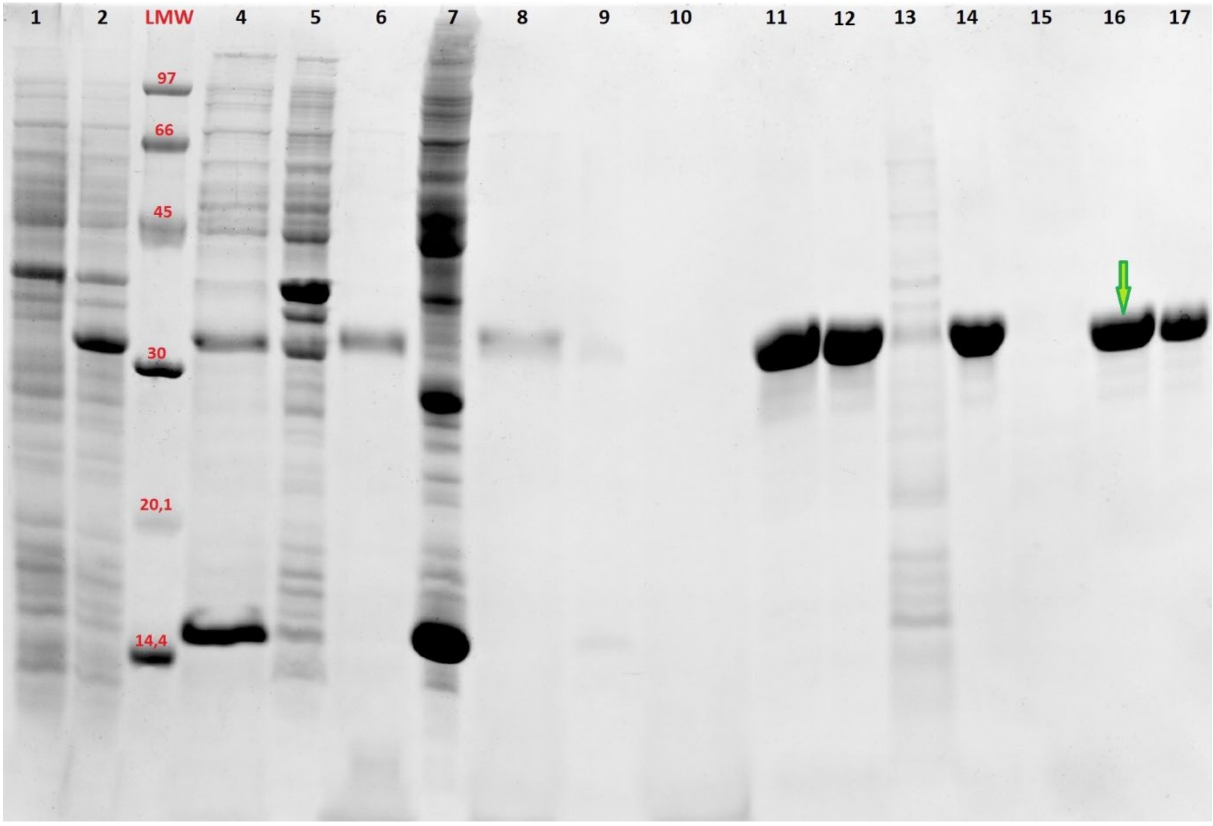

1 - bacterial culture (BLR (DE3) / RE15mR) medium before induction

2 – bacterial culture medium 4 hours after IPTG induction

3 – protein molecular weight marker LMW

4 - supernatant after bacterial cell sonication and centrifugation

5 - protein precipitate from bacterial cells after sonication and centrifugation

6 - supernatant after proteins denaturation at 90 °C and centrifugation

7 - precipitate after proteins denaturation at 90 °C and centrifugation

8 - sample supernatant from ammonium sulphate desalting to 20% after centrifugation

9 - sample precipitate from ammonium sulphate desalting to 20% after centrifugation

10 - sample supernatant from ammonium sulphate desalting 20-40% after centrifugation

11 - sample precipitate from ammonium sulphate desalting 20-40% after centrifugation

12 - sample supernatant after dialysis into 20mM Tris buffer and centrifugation

13 - sample supernatant after dialysis into 20mm Tris buffer and centrifugation

14 - Macro-Prep High Q column purification - unbound proteins

15 - 0.5M salt solution from Macro-Prep High Q resin washing

16 - Final protein, after dialysis to water, filtered (20 µl)

17 - Final protein, after dialysis to water, filtered (10 ul)
